# Cellular basis for blade splitting in bull kelp, *Nereocystis luetkeana*

**DOI:** 10.1002/ajb2.70262

**Published:** 2026-09-08

**Authors:** Alana K. Breitkreutz, Patrick T. Martone

**Affiliations:** ^1^ Department of Botany The University of British Columbia Vancouver V6T 1Z4 BC Canada

**Keywords:** biomechanics, dehiscence, development, histology, lamina, Laminariales, morphogenesis, Phaeophyceae, programmed cell death

## Abstract

**Premise:**

Blade splitting in bull kelp, *Nereocystis luetkeana* is central to thallus development and function, yet the cellular basis of this process remains poorly understood. Two historical models proposed contrasting mechanisms: One attributes blade separation primarily to programmed cell death, whereas the other suggests mechanical tearing followed by tissue regeneration. Resolving the mechanism underlying blade splitting is important for understanding kelp morphogenesis and functional morphology in high‐flow environments.

**Methods:**

We used bright‐field light microscopy to examine histological sections of developing blades undergoing separation and compared their tissue structure with predictions from the two historical models. Cellular organization, tissue thickness, and evidence of cell death and recovery were assessed during successive stages of blade separation.

**Results:**

We found no evidence of programmed cell death associated with blade division. Instead, blade splitting proceeds through a developmental sequence involving localized tissue thinning, mechanical tearing, and rapid healing. Thinning occurs without a loss of cells, indicating reduced extracellular matrix rather than cell death. Following mechanical tearing of the blade tissue, newly exposed blade margins rapidly undergo cell proliferation to regenerate new blade surfaces. Comparisons of this process to sorus detachment in this species, which involves programmed cell death, further demonstrate that blade splitting operates through a distinct physiological pathway.

**Conclusions:**

Blade splitting in *Nereocystis* is driven by mechanical tearing facilitated by localized reductions in blade thickness rather than programmed cell death. By subtly modifying tissue geometry, developing blades harness hydrodynamic forces to direct tearing and thereby produce streamlined morphologies and extensive habitat in hydrodynamically stressful environments.

Along the northeastern Pacific coast, floating canopies of *Nereocystis luetkeana* (K. Mertens) Postels & Ruprecht (Phaeophyceae, Laminariales) form a dominant source of habitat and refuge for coastal fishes and invertebrates (Springer et al., 2011; Hondolero and Edwards, 2017; Teagle et al., 2017; Edwards, 2025; Farrer, 2025). These canopies are formed from clusters of long, ribbon‐like blades, all of which originate from a single initial blade that repeatedly divides along its length (MacMillan, 1899; Wells, 1910; Frye, 1906, 1930; Fritsch, 1945; Nicholson, 1970). Longitudinal blade splitting is central to bull kelp morphology and canopy development. Blade splitting determines the number and arrangement of blades within the canopy, thereby influencing how thalli interact with moving water. These morphological traits affect both the photosynthetic surface area of the kelp and the hydrodynamic forces acting upon it. Despite the ecological importance of this kelp, the cellular and developmental basis for blade splitting remains poorly understood. Here, we examine the histological basis of blade separation to clarify how this foundational species generates the structures that support the surrounding ecosystem.

Blade splitting in the bull kelp is associated with long, linear tissue features known as lines of dehiscence (LOD), which often develop before splits or extend distally from the apex of an ongoing division (Wells, 1910; Fritsch, 1945; Nicholson, 1970). These features mark regions of altered tissue that indicate sites of future or active blade separation and provide a valuable opportunity to examine early stages of blade splitting (Wells, 1910).

Two contradictory models of blade separation in *Nereocystis* have persisted since the turn of the 20^th^ century (MacMillan, 1899; Wells, 1910). Both existing models are based on histological series traced along LOD, starting distally and moving through the apex of an extending split to the point where the two new blades are fully formed (see Figure 1). Each proposes a distinct mechanism by which new blades arise from a growing thallus. These models were recognized as incompatible even at the time of publication (Wells, 1910; Fritsch, 1945; Walker, 1980). Both models have influenced subsequent interpretations of *Nereocystis* development, including those of Fritsch (1945), yet no study has empirically tested the predictions of either model.

**Figure 1:**
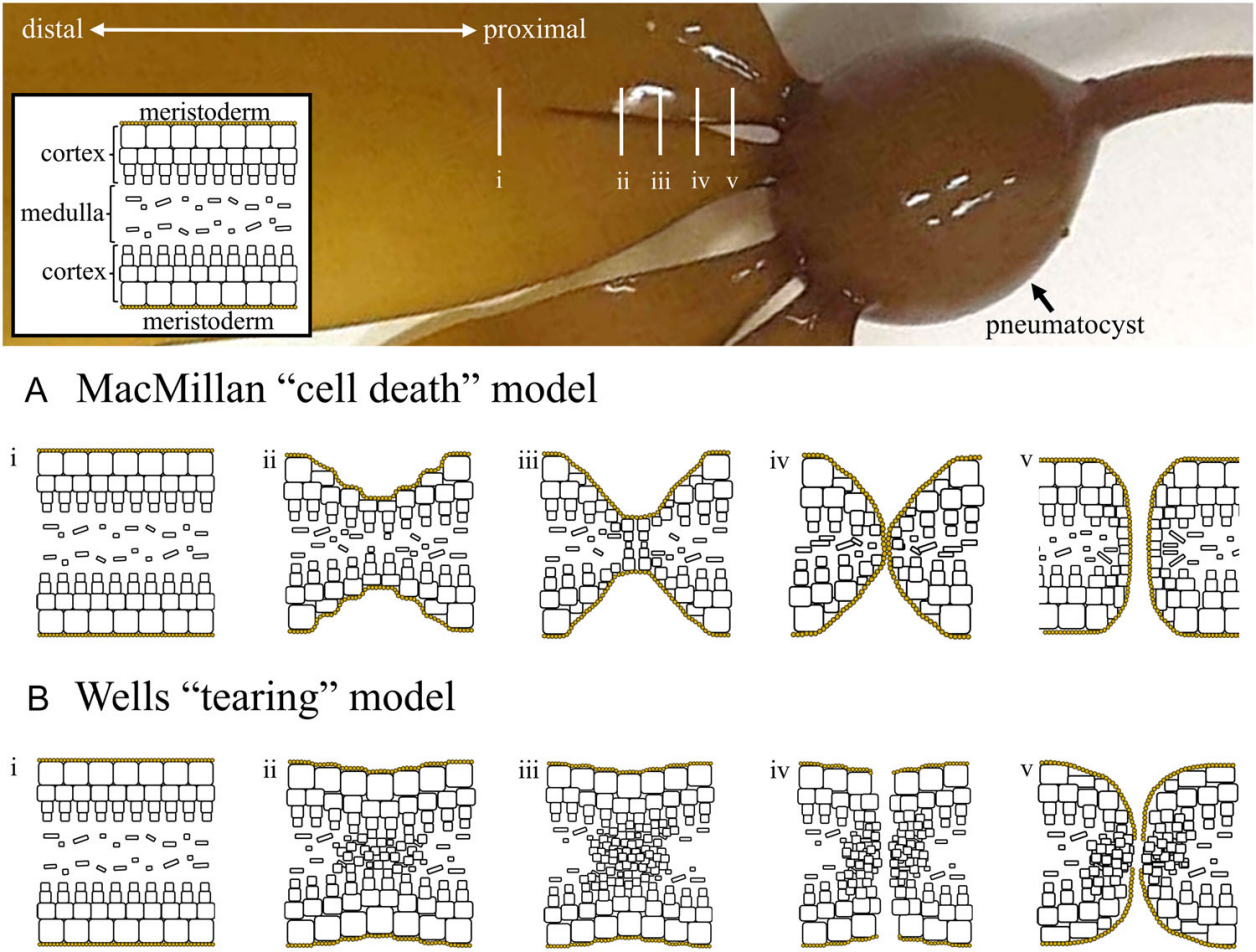
Two historical models of blade division in *Nereocystis luetkeana*. Direction and approximate location of the histological series are shown on a juvenile *Nereocystis* with an extending split. Distal–proximal axis is indicated relative to the pneumatocyst. Histological series are arranged from early to late stages of tissue modification (i–v), whereas blade separation proceeds in the opposite direction (v–i). Inset: Schematic cross section of a nondividing blade showing the meristoderm, cortex, and medulla cell layers. (A) MacMillan “cell death” model (1899). Inner tissues deliquesce and disintegrate, causing the blade to pinch inward as the meristoderm folds to form the new blade surface before separation. (B) Wells “tearing” model (1910). Internal changes weaken the blade before tearing‐based separation; new surfaces form by regrowth along the tear margins.

MacMillan (1899) proposed that each blade split begins with the deliquescence of inner cortical cells (see Figure 1A). As internal cells die and disintegrate, the inner tissue layer thins, causing the overlying meristoderm—the outermost pigmented layer of the kelp blade—to pinch inward and outline the boundary of the new blades. In this model, the edges of new blades are fully formed before any physical separation of the two laminae occurs. Mechanical tearing may later split the two surfaces at the pinch point, but the primary initiating events are programmed cell death and localized inward folding of the meristoderm (see Figure 1A). This model bears similarities to leaf division in *Monstera* (Gunawardena et al., 2005) and the lace plant (Gunawardena et al., 2004). Programmed cell death (PCD) has been documented during soral detachment in *Nereocystis* (Walker, 1980), but whether PCD is involved in blade splitting remains heretofore unknown but nevertheless, past examination of cell death during soral detachment provides a useful comparison for interpreting cellular damage and PCD during blade splitting.

Wells (1910) argued that internal tissue changes—specifically the proliferation of cortical cells—produced new tissue surrounded by older stronger tissue, thereby weakening the thallus along predictable lines without immediately giving rise to new blades (Figure 1B). Instead, mechanical tearing, often caused by wave action, underlies separation. Regrowth along the tear margins then produces the surface of each new blade. In Wells’ model, the new surface forms after the physical rupture (see Figure 1B).

These two models differ in the structural features they predict. In MacMillan's model, the meristoderm remains continuous throughout separation, and the underlying cortex and medulla are never exposed. As cells in the inner tissues die and disintegrate, one would expect to see signs of PCD, such as broken or deformed cell walls, bacterial invasion, or loss of characteristic staining properties (see Walker, 1980). In addition, the cell number and the total thickness of cell walls in cross section would decrease ahead of a split. In Wells’ model, tearing disrupts the continuity of the meristoderm, leaving the cortex and medulla exposed along the tear margin prior to healing. Because the model proposes that cortical cell proliferation is the source of tissue weakness, cell number and total cell wall thickness in cross section would both increase ahead of a split. These morphological differences (illustrated in Figure 1) provide potential diagnostic features to evaluate which model more accurately reflects blade formation in *Nereocystis*.

To improve our understanding of blade splitting and blade development in *Nereocystis*, we compared the two models of blade separation by looking for the anatomical and cellular features that each predict. To that end, we analyzed histological sections of laminae that were actively splitting, spanning developmental stages from LOD on initial single blades to higher‐order splits separating mature blades. We also compared naturally split blades to manually torn ones to distinguish cellular features involved in separation from those involved in healing. Together, these observations provide a framework to refine, reconcile, and expand existing models of blade separation in *Nereocystis* and to better understand the formation of bull kelp canopies.

## MATERIALS AND METHODS

Blades from approximately 70 individual *Nereocystis* thalli were collected over three summers (2021–2023) from Stanley Park (Vancouver, BC, Canada) and Ogden Point (Victoria, BC, Canada). From each thallus, one blade with signs of active blade division was selected. These included blades with (1) a visible LOD, (2) an actively extending split with an LOD, or (3) an actively extending split without a clearly visible LOD. A small number of intact blades without visible splitting features were also processed as histological controls. Blade tissue was excised to encompass the region of interest associated with blade division. In blades with an extending split but no associated LOD, tissue was collected from about 5 mm proximal to the split apex and 10 mm distal. In blades with a visible LOD, tissue was collected along the length of the LOD and up to 10 mm distally. Excised tissues included approximately 2 mm of tissue on either side of the region of interest and were preserved in 5% v/v formalin–seawater before processing.

From each tissue sample, 3 × 15 mm pieces were selected to capture different positions along an extending split or LOD, representing different stages of development. In total, approximately 100 tissue pieces were dehydrated through a graded ethanol series (2.5%, 5%, 7.5%, 10%, 15%, 20%, 30%, 50%, and 75% for 30 min each, 100% overnight). After dehydration, samples were infiltrated with LR White acrylic resin (MilliporeSigma Canada, Oakville, ON, Canada) in a series with a decreasing volume of ethanol (1:1 for 4 h, 3:1 for 4 h, 100% LR White overnight). The next day, samples were placed into fresh resin for 2 h, then into gelatin capsules containing fresh resin, which was polymerized in an oven at 62°C for 8 h. A Sorvall MT‐2B ultramicrotome and glass knife were used to cut serial cross sections (8–10 µm thick) from tissue distal to an extending split (often along an LOD) to within the extending split (Figure 1). All sections were stained with 0.1% w/v toluidine blue in water, known to highlight cell walls and other polysaccharide‐rich structures. Additional staining was carried out with safranin O (1% w/v safranin O/water) and vanillin–HCl (10% vanillin in a freshly prepared 2:1 mixture of 95% ethanol and concentrated hydrochloric acid). Sections were viewed using bright‐field optics and an Olympus BX51WI microscope and imaged with an Olympus DP21 digital camera (Olympus, Tokyo, Japan).

**Figure 2:**
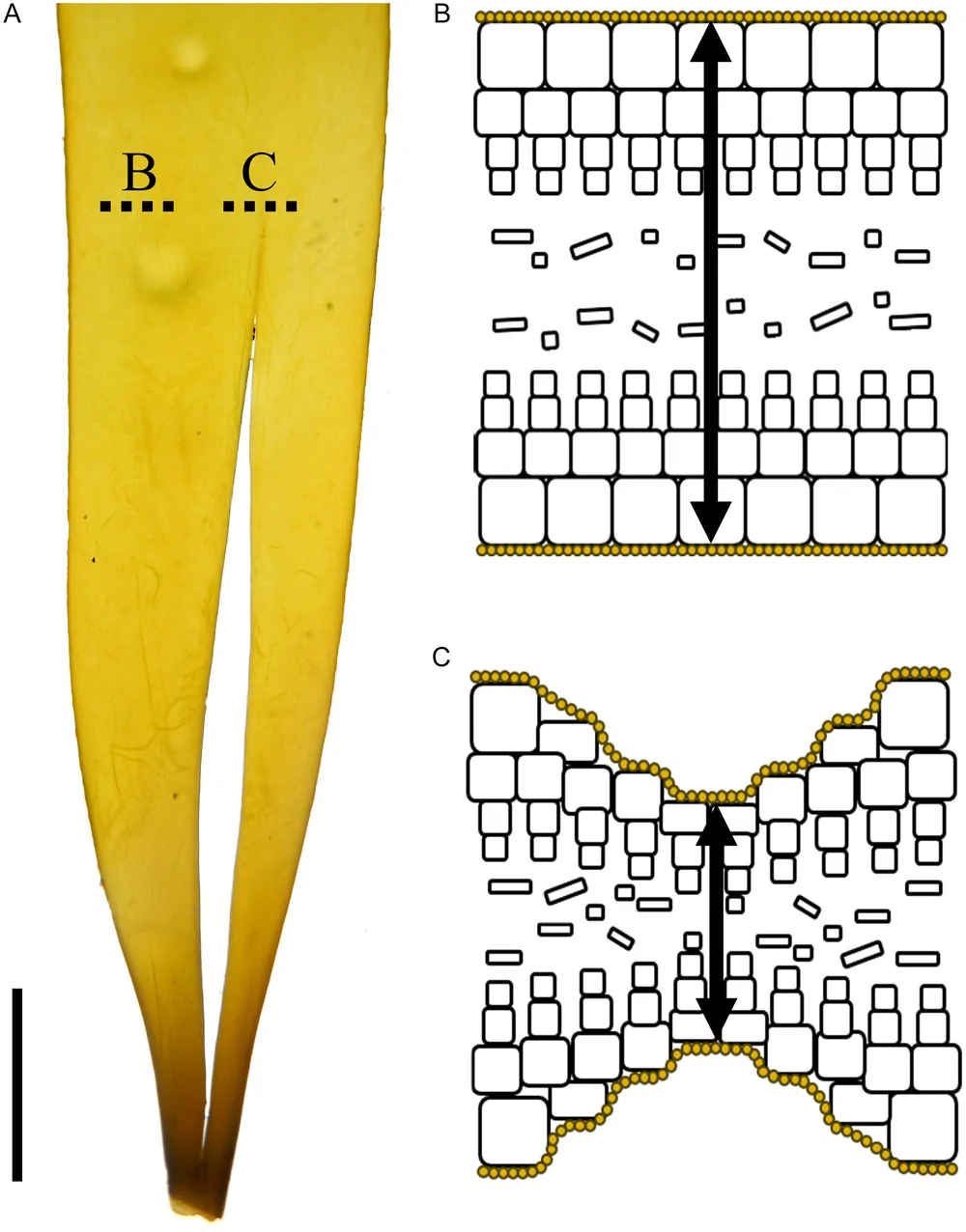
Sampling scheme for comparing tissue features in cross sections of blades of *Nereocystis luetkeana*. (A) Blade with an extending split showing sampling scheme for comparing tissue structure between (B) nonsplitting tissue and (C) tissue distal to an extending split. Blade thickness, cell counts, and cumulative cell‐wall thickness were measured across the cross sections (locations shown by double‐headed arrows). Scale bar (bottom left, for all panels) = 1 cm.

To distinguish tearing‐related tissue modification from PCD, we examined cortical and medullary cells for structural and staining changes documented in sori, including loss of toluidine blue affinity, degradation of cell walls, and breakdown of cortical organization. To evaluate differences in tissue structure between nonsplitting regions and tissue ahead of a developing split (Figure 2), blade thickness, cell number, and the cumulative thickness of all cell walls were measured along a single transect perpendicular to the blade surface (*N* = 33 paired samples) and analyzed with paired *t*‐tests. Data were analyzed using R version 4.4.1 (R Core Team, 2024).

To distinguish tissue changes associated with wound healing from those associated with blade splitting, we generated longitudinal wounds that were not part of the natural splitting process. Three blades were excised from seven thalli at Stanley Park, Vancouver, BC. To examine wound healing around a tear, we used a razor to make a 1‐mm incision in one blade from each trio and was then extended it longitudinally by manual tearing. To examine healing around any longitudinal cut, we similarly cut a second blade. A third blade was left untreated. Blades were maintained in a tumble tank for 4 days to allow healing.

After 4 days, a tissue piece encompassing the wound apex, approximately 5 mm of tissue bth distal and proximal to the apex, and approximately 2 mm of tissue on either side of the wound, was excised and fixed in LR White resin, as described above. A tissue piece of the same dimensions was excised from each untreated control blade at the corresponding position relative to the pneumatocyst. Serial sections were then cut starting distal to the wound apex and progressing into the wound, in the same sampling orientation as the series taken from naturally extending splits. Sections were stained with toluidine blue and safranin O, as described above. The histology of these healing‐only wounds was compared with that of the series obtained distal to naturally extending splits.

## RESULTS

**Figure 3:**
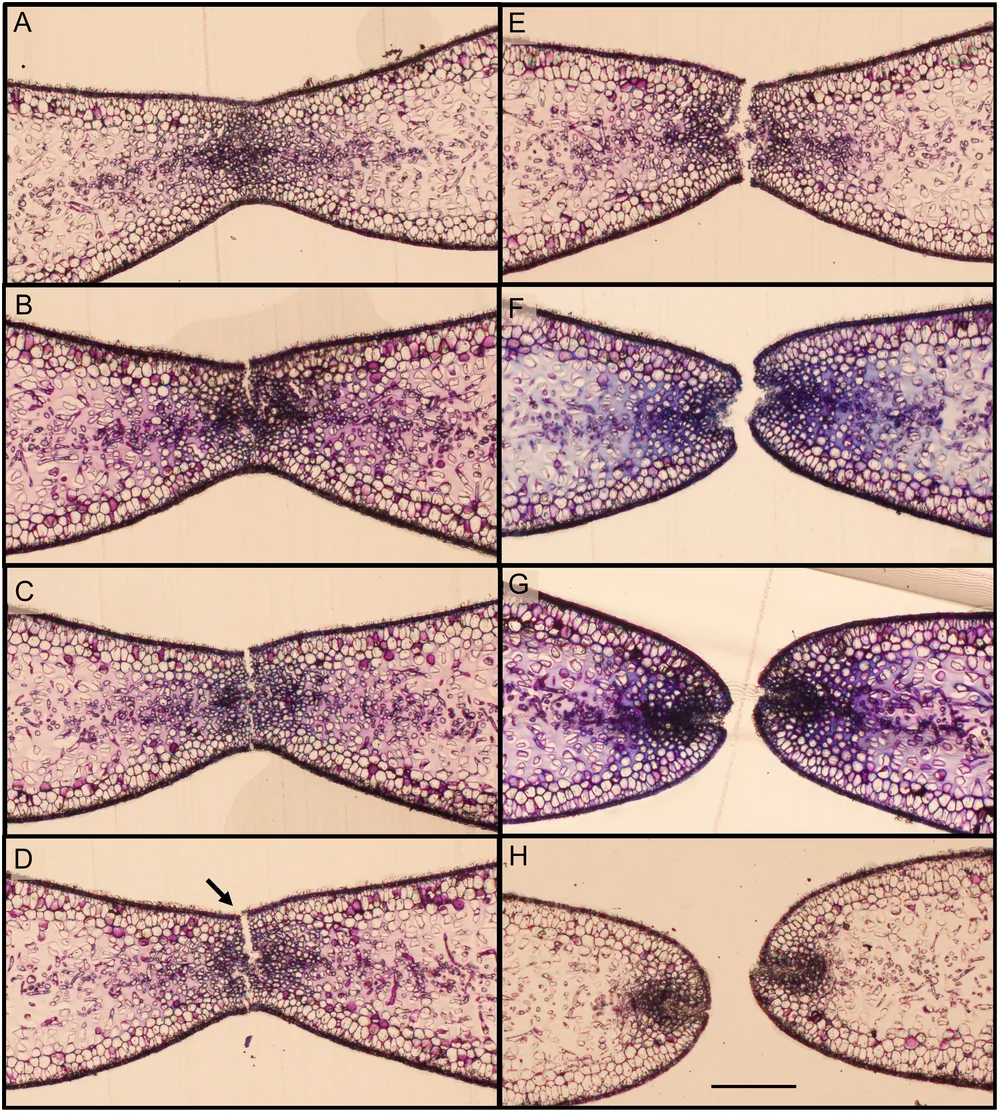
Serial sections of splitting blade of *Nereocystis luetkeana* showing tissue modification, tearing, and healing. Sections progress proximally from just distal to the apex of an extending split. (A) Thinned blade tissue distal to the end of an extending split. (B) Initial separation and discontinuities in the meristoderm. (C) Continued separation exposing inner cortical and medullary tissues. (D) Broken cells at the exposed inner tissue surface (arrow). (E) Complete separation of the blade from top to bottom, with exposed inner tissues and discontinuous meristoderm on the two new blade surfaces. (F) Early stages of healing. (G) As healing progresses, cortical cell division fills in the new blade surface, and the top and bottom meristoderm grow toward each other. (H) Advanced healing with extensive cortical cell division restoring the blade cross section. Scale bar (bottom right, for all panels) = 200 µm.

Blade tissue distal to an extending split (e.g., Figure 1: ii–iii) was thinner than surrounding tissues (Figure 3A), with an average reduction in thickness of approximately 33.5% (340.0 ± 29.7 µm distal to splits vs. 511.0 ± 39.4 µm in adjacent, nonsplitting tissue; paired *t*‐test, *t* = 9.37, df = 32, *P* < 0.001). Cortical and medullary cells retained normal staining patterns and intact cell walls. Despite the reduction in thickness, the number of cells spanning the blade cross sections did not differ between the tissue distal to an extending split (13.7 ± 0.8 cells) and adjacent, nonsplitting tissue (13.5 ± 0.8 cells; paired *t*‐test, *t* = –0.82, df = 32, *P* = 0.42). Similarly, cumulative cell wall thickness in the cross sections did not differ between the region distal to an extending split (74.9 ± 8.6 µm) and adjacent, non‐splitting tissue (73.7 ± 8.2 µm; paired *t*‐test, *t* = –0.54, df = 32, *P* = 0.59).

At the site of blade separation (e.g., Figure 1: iv), top and bottom meristoderm layers were not connected and cortex and medullary cells were exposed at the edge of the two new blades (Figure 3B–E). A small number of cells of the exposed inner tissues, on the immediate surface of the two new blades, appeared to be broken open (Figure 3D, indicated by arrow). None of the broken cells lost their characteristic staining with toluidine blue.

Immediately proximal to the apex of the split (e.g., Figure 1: v), signs of rapid healing were observed, with the meristoderm growing over the exposed cortex and medulla (Figure 3F, G). Extensive cell division occurred within the cortex to gradually restore the normal blade cross section (Figure 3H). A slight overlap between the top and bottom meristoderm layers (see Figure 3H) was usually present in healing blade edges.

**Figure 4:**
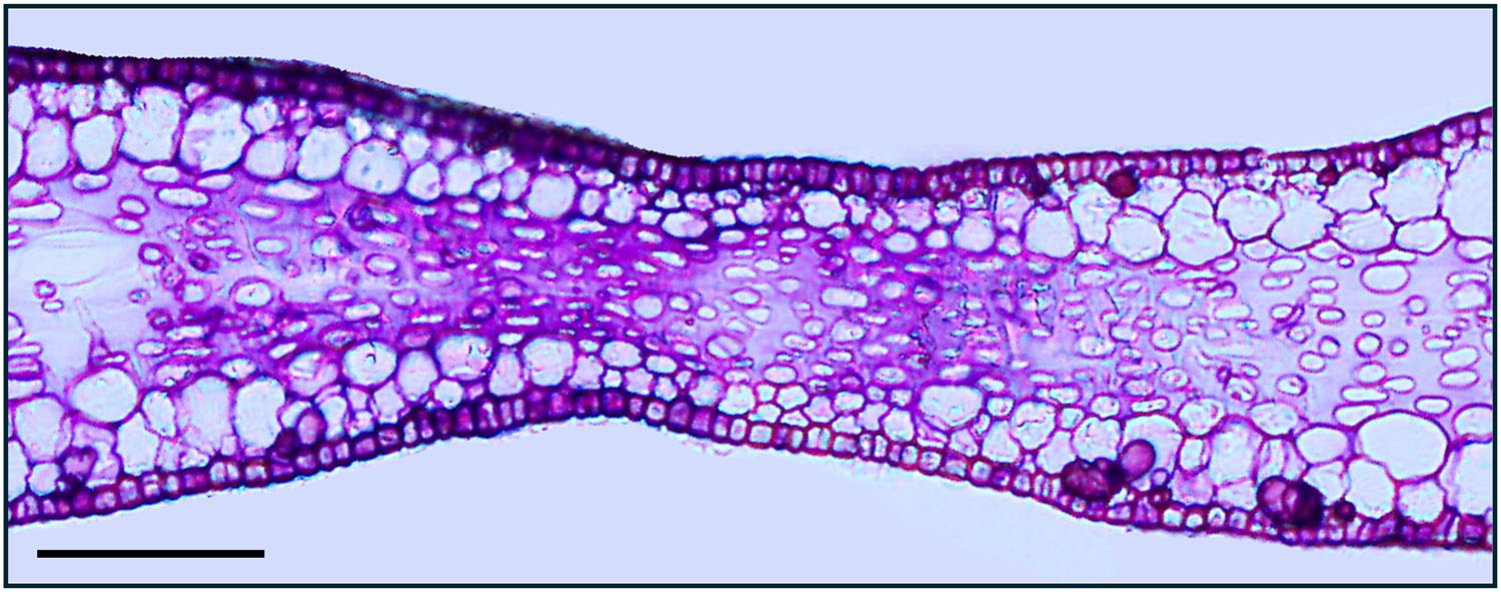
Cross section of blade tissue distal to an actively extending split. Note increased staining intensity of extracellular matrix with toluidine blue and marked thinning. For clarity, ImageJ was used to replace the background with a uniform color; no tissue pixels were altered. The original, unedited image is in Appendix S1: Figure S1. Scale bar = 100 µm.

**Figure 5:**
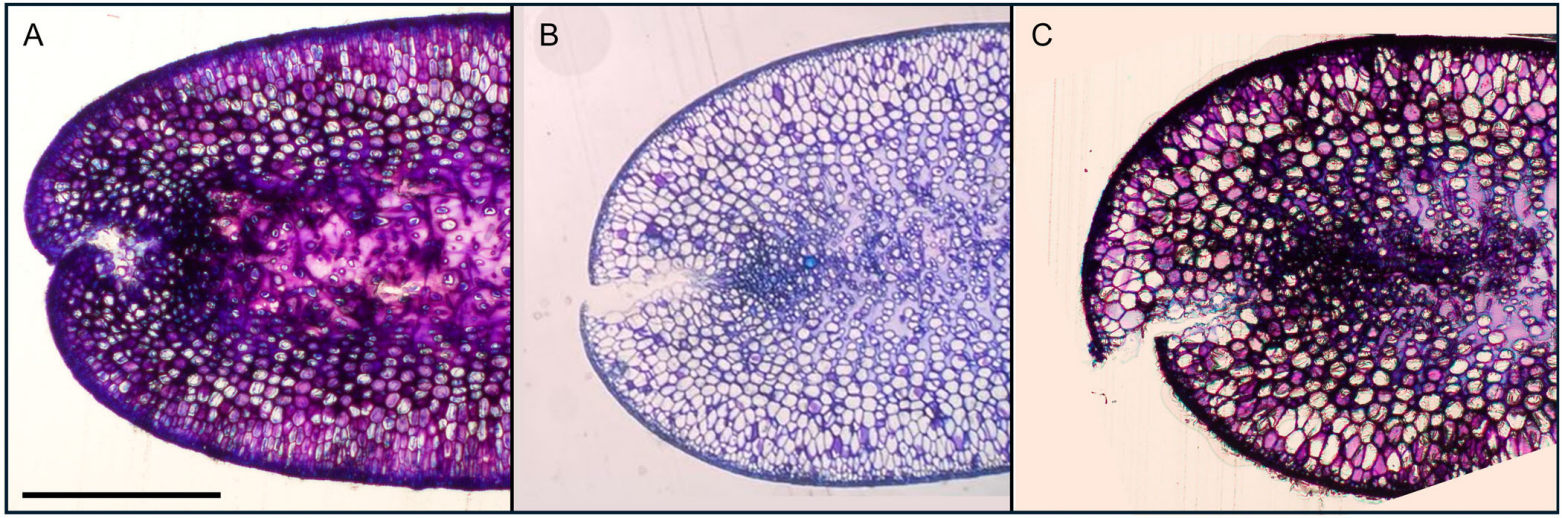
Three newly formed blade edges after splitting. Blade edge (A) proximal to the apex of a naturally extending split, (B) after a manual tear was allowed to heal for 4 days, and (C) after a razor incision was allowed to heal for 4 days. Note the dark toluidine blue stain where the wound is healing in all three blades. Scale bar = 400 µm.

Sections distal to an advancing split consistently showed dark extracellular staining with toluidine blue (Figure 4). Safranin O staining was also more intense in these regions (Appendix S1: Figure S2). Vanillin–HCl did not stain the extracellular space. Sections at the distal edge of a manually introduced longitudinal tear were morphologically similar to those observed in naturally occurring splits (Figure 5A, B). Increased staining with toluidine blue was present in the medulla near the healing edge, and division of meristoderm and cortical cells forming the new blade surface resembled the healing process of a natural extending split. Blades healing from a razor incision had the same features as the manual tears (Figure 5C).

## DISCUSSION

### An updated model of blade splitting

Our re‐examination of blade division in *Nereocystis luetkeana* revealed a consistent sequence of events: localized tissue modification, mechanical tearing, and rapid healing. This sequence was often marked by the appearance of a visible LOD, but even in the absence of a fully formed LOD, structural changes were detected in the tissue immediately distal to an extending split.

Before the natural splitting, blade tissue becomes thinner along a longitudinal axis that aligns with the anticipated path of blade separation. Along this axis, the extracellular matrix shows increased staining intensity with toluidine blue and safranin O, suggesting a chemical shift involving elevated levels of sulfated or acidic polysaccharides alongside structural modification. When a visible LOD is present, shifts in thickness and staining intensity can begin as much as 30 cm distal to the extending split and become more pronounced moving proximally. None of the hallmarks of soral‐margin degradation (see Walker, 1980) were present along the LOD or in tissue distal to extending splits. Although tissue thickness is reduced along the LOD, cell number remains unchanged, indicating that thinning is primarily driven by reduced extracellular matrix rather than cell death. Localized thinning likely has a direct effect on both bending and stress concentration in splitting blades (Breitkreutz and Martone, 2026), and, indeed, our present study shows that blade separation ultimately occurs through tearing. Discontinuity between the upper and lower meristoderm, along with consistent exposure of internal cortical and medullary tissues at the split apex, points to a tearing‐based mechanism. These features were consistently observed at any distance from the pneumatocyst and in both mature and developing blades.

Healing begins immediately after tearing and involves both morphological and chemical changes. Cortical cell proliferation and meristoderm expansion rapidly rebuild the wound surface of the new blades. A slight overlap in the top and bottom meristoderm is frequently observed at healing margins, suggesting directed growth over exposed inner tissues. Increased staining intensity with toluidine blue and safranin O in healing regions points to a localized chemical change, potentially involving matrix components that facilitate healing. Importantly, the same pattern of cortical cell proliferation, meristoderm regrowth, and increased staining intensity was observed in manually torn blades, suggesting that chemical changes involved in healing are activated specifically by tissue disruption, whether developmentally programmed or induced by an external force.

### Reconciling two historical models

The histological investigation presented here strongly supports several of Wells’ (1910) propositions, particularly his proposal that mechanical tearing plays a central role in the separation process. Consistent with his observations, discontinuity of the meristoderm and exposure of internal blade tissues appear at the apex of every extending split. However, our interpretation of cortical cell proliferation differs. Wells (1910) proposed that proliferation contributes to the splitting process by altering the tearing properties of the tissue. In contrast, our results indicate that cortical cell proliferation occurs after tearing has taken place, as part of a localized healing response. Tissue immediately adjacent to fresh tears shows a greater density of cortical cells only on a newly forming blade surface, supporting the view that proliferation aids blade reconstruction rather than promoting division.

Consistent with MacMillan's (1899) model, tissue modification preceding splits does involve thinning of the blade. However, this thinning does not appear to result from cell disintegration. Rather, it reflects differences in extracellular matrix deposition. Furthermore, healing splits can superficially resemble MacMillan's drawings of “cell gelatinization” followed by inward pinching of the meristoderm. In practice, though, the top and bottom meristoderm of a new blade surface come into contact only after the initial separation has already occurred. MacMillan (1899) may have inferred the process backward from the healing stage or may have interpreted an extreme thickness differential—where tissue on either side of the split thickened substantially compared to thinner tissue along the split's path—as evidence of cell death.

### A self‐perpetuating developmental program

Across all extending splits examined, whether or not a visible LOD was present, tissue immediately distal to the split apex showed increased staining intensity with toluidine blue and in most cases was markedly thinner. In these cases, the split has propagated beyond the end of the original LOD, or the LOD in that region was too short or subtle to detect macroscopically. Thus, LOD “visibility” largely reflects LOD length relative to the current position of the split apex, not an on–off state of the modification itself.

Interestingly, when splits propagate beyond their initial LOD, or when they arise in regions where no visible LOD has yet formed, the tissue immediately ahead of the crack nevertheless shows the same chemical and structural signatures characteristic of LOD. These observations imply a positive‐feedback mechanism: Once tearing is initiated, the tissue immediately ahead of the crack tip begins to undergo the same matrix modification and thinning observed along the established LOD. In this view, the LOD is not a fixed, predetermined structure but a dynamic, advancing zone of weakened tissue that develops in synchrony with crack propagation. This dynamic feature ensures continued extension of the split without requiring a pre‐existing LOD.

Tissue modification (and eventually LOD formation) may be driven by changes in hormone signaling. Damage near the basal meristem (i.e., the blade's petiole) has been shown in other kelps to disrupt hormonal cues and induce developmental changes distally, such as sorus formation downstream of hole punches (Buchholz and Lüning, 1999; Kai et al., 2006). This type of hormonal regulation is similar to branching in terrestrial plants, which is inhibited by auxins emanating from apical meristems and only increases when meristems are removed or have grown sufficiently far away (Thimann and Skoog, 1933; Cline, 1991). A similar signaling shift could be caused by a splitting (“damaged”) blade, inducing matrix modification distally for an LOD and creating a longitudinal path for the split to propagate. Under this framework, blade splitting in *Nereocystis* may reflect a coupled mechanical–physiological system: environmental forces initiate or accelerate tearing, and blades respond by modifying tissue properties ahead of each crack to guide its trajectory and ensure successful healing. Future work could empirically test these ideas by manually introducing a longitudinal split in nonsplitting blades and observing whether such damage induces an LOD.

Ultimately, rather than resist mechanical failure, *Nereocystis* blades promote it, directing damage along lines that will divide photosynthetic tissues into narrower blades that experience less drag and cluster more effectively in high‐flow environments (Breitkreutz, 2026). Flow reinforces this mechanistic link by accelerating split propagation (Breitkreutz and Martone, 2026). In this way, *Nereocystis* harnesses mechanical failure into a morphogenetic mechanism. No dedicated organ or enzymatic abscission zone is required; instead, simple geometric changes permit blades to reshape through interactions with the hydrodynamic environment.

## AUTHOR CONTRIBUTIONS

A.K.B. planned and designed the research under supervision from P.T.M. A.K.B. did the research including collecting and analyzing the data. A.K.B. and P.T.M. interpreted the data. A.K.B. wrote the manuscript. P.T.M. helped edit the manuscript.

## Supporting information


**Appendix S1:** Supporting Figures S1 and S2. Light micrographs of blades of Nereocystis luetkeana.
**Figure S1:** Raw, unedited version of Figure 4 showing the same region of tissue distal to the apex of an extending split, with increased toluidine blue staining in the extracellular space.
**Figure S2:** Blade cross section after being manually torn and allowed to heal.

## Data Availability

All data and scripts are available online at github.com/martonelab/nereocystis-blade-splitting.
