# Supplementary material for: Cellular basis for blade splitting in bull kelp, Nereocystis luetkeana
Source: Am J Bot. 2026 Sep 8;113(9):e70262. doi: 10.1002/ajb2.70262 (PMC13595501; doi:10.1002/ajb2.70262)
Supplement: Supplementary file 1 [file AJB2-113-e70262-s001.docx]

**Appendix S1.** Supporting Figures S1 and S2. Light micrographs of cross sections of blades of *Nereocystis luetkeana*. Bright-field optics.

**Figure S1.** Original, unedited version of Figure 4 showing the same region of tissue distal to the apex of an extending split, with increased toluidine blue staining in the extracellular space. Scale = 100 µm.

**Figure S2.** Blade cross section after being manually torn and allowed to heal for 4 days. Top panel: toluidine blue stain. Bottom panel: safranin O stain. Scale = 200 µm.
